# Chagas’ Disease: An Emergent Urban Zoonosis. The Caracas Valley (Venezuela) as an Epidemiological Model

**DOI:** 10.3389/fpubh.2014.00265

**Published:** 2014-12-03

**Authors:** Servio Urdaneta-Morales

**Affiliations:** ^1^Laboratory for the Biology of Vectors and Parasites, Tropical Zoology and Ecology Institute, Central University of Venezuela, Caracas, Venezuela

**Keywords:** Chagas’ disease, emerging urban zoonosis, Caracas Valley (Venezuela)

## Abstract

The unprecedented emergence of important public health and veterinary zoonoses is usually a result of exponential population growth and globalization of human activities. I characterized Chagas’ disease as an emergent zoonosis in the Caracas Valley (Venezuela) due to the following findings: the presence of reservoirs (*Didelphis marsupialis*, *Rattus rattus*) and vectors (*Panstrongylus geniculatus*, *Panstrongylus rufotuberculatus*) infected with *Trypanosoma cruzi* in urbanized or marginalized areas; the elevated contact between *P. geniculatus* and human beings detected by parasitological and molecular examinations of triatomine feces demonstrated the possibility of transmission risks; a study of outbreaks of urban Chagas’ disease reported the first proven case of oral transmission of *T. cruzi* to human beings; the risk of transmission of glandular metacyclic stages from marsupials by experimental ocular and oral instillation; mice genitalia infected with *T. cruzi* contaminated blood resulted in the formation of amastigotes very close to the lumen suggesting that there may be a possibility of infection via their release into the urine and thence to the exterior; the ubiquitous histotropism and histopathology of *T. cruzi* was demonstrated using a mouse model; the presence of experimental *T. cruzi* pseudocysts in adipose, bone-cartilage, and eye tissue indicated a potential risk for transplants. Socio-sanitary programs that include improvements in housing, vector control, and access to medical treatment, as well as strategies aimed at combating social inequalities, poverty, and underdevelopment should be undertaken in those areas where zoonoses are most prevalent. Disciplines, such as Ecology, Epidemiology, Medical Entomology, Human and Veterinary Medicine, Environmental Studies, Public Health, Social and Political Studies, Immunology, Microbiology, and Pharmacology could all provide important contributions that aim to reduce the occurrence of factors governing the spread of emergent diseases.

## Introduction

American trypanosomiasis (Chagas’ disease), a metaxenic zoonosis caused by *Trypanosoma (Schizotrypanum) cruzi* Chagas, 1909, is endemic to Neotropical and Nearctic regions (Salt Lake City, 41° N in USA/56° S in the argentine Patagonia and northern Chile as well as the Caribbean Islands). A total of 8 million people in 21 countries are infected by the disease with mortality rates of approximately 10,000 per year. This parasitosis is found in particular landscapes inhabited by mammal reservoirs and insect vectors (Hemiptera, Reduviidae, Triatominae), which together make up the wild cycle, the most primitive zoonotic transmission dynamic, in which the parasite travels between more than 200 species of mammals from seven orders and about 140 species of insects in 15 genera (1, 2).

The most ancient reservoirs in this cycle are the Eutherian (Cingulata, Dasypodidae: armadillo); Rodentia (Echimyidae); and Metatherian (Didelphimorphia, Didelphidae: opossum) (3, 4). These synanthropic mammals have migrated from their natural ecosystems to human communities, while at the same time, human beings have encroached onto their habitats. In both cases, *T. cruzi* infects individuals giving rise to the so-called peridomestic and domestic Chagas’ disease cycles. This accidental anthropozoonosis may develop in rural as well as urban areas and is conditioned by both environmental and social elements (1, 5). In Venezuela, *T. cruzi* circulates between 39 species in the Marsupiala, Edentata = Xenartra, Chiroptera, Carnivora, Rodentia, Primates, and Lagomorpha and is transmitted by 22 insect vectors. The main vector is *Rhodnius prolixus* due to its broad distribution (over nearly three quarters of the total land area of Venezuela) and its high domiciliation (6–8).

*Trypanosoma cruzi* is a hemoflagellate (Protozoa, Kinetoplastida, Trypanosomatidae) with two developmental stages: extracellular bloodstream trypomastigotes and intracellular tissue amastigotes. In vectors, both forms are extracellular: epimastigotes that develop in the lumen of the midgut, and metacyclic trypanosomes found in the rectal part of the hindgut. The latter of these two forms is the infective stage, and transmission of the parasite primarily occurs when contaminated feces evacuated by the parasitized insect come into contact with healthy mucosa or the damaged skin of the host. In contrast, in the African trypanosomes and the Neotropical *Trypanosoma rangeli*, the parasites initially develop in the gastrointestinal tract of the insect vector and from there migrate by tropism to the salivary glands where the infective stages develop before being transmitted mechanically when the insect bites a host. In both cases, the *Trypanosoma* species cause zoonotic parasitosis in human beings (9, 10).

American trypanosomiasis discovered in Brazil by Chagas (11), was demonstrated in Venezuelan human beings by Tejera (12), and Pifano (13) described the characteristics of the environmental niches of this zoonosis as well as the features of the principal bioregions where it develops.

## Definition of Emerging Infectious Diseases and Zoonoses

The emergence and spread of smallpox during the “discovery” of America in the 16th century, which attacked vulnerable non-immune indigenous populations and the sudden appearance of the great pandemics: bubonic plague, cholera, typhus, syphilis, and leprosy in Europe during both the Middle Ages and the Modern period are all examples of emerging diseases. These were triggered by massive migrations from rural to more-developed areas resulting in overcrowding and social changes, which together with a lack of hygiene exposed populations to transmissible agents (14).

Among the many, and often divergent, definitions of the concept of emergent infectious diseases, two appear to be the most appropriate as they are based on the dynamics of several elements that act synergistically to produce the diseases. The first of these is “Infectious diseases are said to be emergent when qualitatively unexpected phenomena resulting from local interactions appear; this tends to occur suddenly” (14). The other is that emerging infectious diseases are infections that have newly appeared in a population, or already exist but whose incidence is rapidly expanding over a geographical, host or vector range, or have been reported in new populations (15–18). Thus, every infectious disease is emergent until it reaches endemic status. These broad definitions encompass a range of human diseases all of which represent a significant threat to public health.

Zoonoses are infectious diseases that are transmitted between their natural hosts and human beings (19).

## Purpose of the Review

The principal determining factors that make up the web of causation of the infectious diseases can be summarized as follows: biological (infectivity and pathogenicity) and molecular properties of the pathogens; characteristics of the vertebrate hosts; vector competence and migration, anthropogenic factors causing significant damage to ecosystems resulting in the alteration or destruction of mammal hosts and insect vectors habitats as well as changes in their behavior; and decrease in the funding allocated to health authorities for their surveillance, prevention, and control. While a few infectious diseases have been eradicated (smallpox) or controlled (dracunculiasis, measles, polio), it is probable that most of the rest of them will not be, at least in the short term, because the causal factors for their emergence remain. This being so and faced with the fact that the recurrence rate will not only continue but also increase, new approaches and tools must be found and implemented in order to at least prevent the establishment of these diseases (10, 15–20). This article was stimulated by these considerations.

The studies based in the Caracas Valley will be compared with reports of emergent diseases produced by zoonotic agents from other countries. The Caracas Valley was selected because it is still classified by the health authorities in Venezuela as “not endemic” and is, therefore, are not included in the Venezuelan National Control Programme (21).

## Characteristics of the Caracas Valley

**Figure 1:**
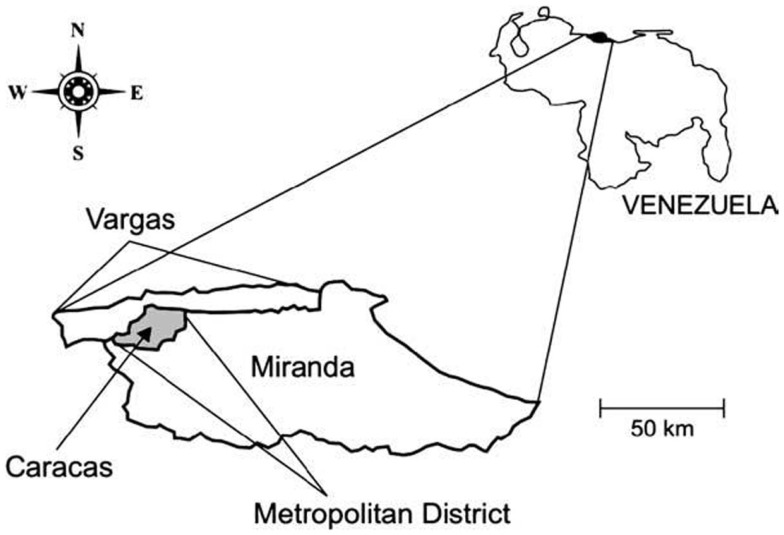
**Map of Venezuela showing the relative location of the area of the Metropolitan District**.

Caracas, the capital city of Venezuela, lies in a depression within the coastal mountain range in north-central Venezuela. The city is located at 960 m.a.s.l. and comprised five municipalities and two federal entities: the Capital district (Libertador municipality) and Miranda state^[]^ (Chacao, Baruta, El Hatillo, and Sucre municipalities). All these municipalities together make up the Caracas Metropolitan district (10° 23′ 18″–10° 34′ 00″ N; 66° 51′ 30″–67° W 10′35″) (Figure 1). The Caracas valley has an altitude of 870–1043 m.a.s.l., average temperature 22°C, and annual rainfall of 870 mm; the natural vegetation is pre-montane forest. The population density of Caracas has been increasing exponentially due to steady migration from other parts of Venezuela, as well as neighboring countries, and is now fully urbanized. This migration has resulted in a population with minimum hygiene standard as well as poor socioeconomic conditions. Much of this population is housed in urban slums, located on the banks of the numerous rivers that cross the city as well as in the neighboring savannas and mountains forming a regrettable belt of misery. The slums adjoin built-up medium and high value residential or commercial areas and similar situations have developed in other areas along the valley, all of which have profoundly modified the natural environment. The Caracas Valley is, thus, an excellent example of the progressive modification of an ecosystem, where an increasing human population has invaded a region already populated by Chagas’ disease insect vectors and mammal hosts (21, 22).

## Chronological Description of Investigations into the Chagas’ Disease Web of Causation in the Caracas Valley That Demonstrate Its Status as an Emerging Urban Zoonosis

The research undertaken by Pifano (22) was the first to demonstrate the presence of Chagas’ disease in the Caracas Valley. This was done by identifying the components of the transmission cycle as follows: reservoirs (*Didelphis marsupialis*) and vectors (*Panstrongylus geniculatus*) captured in slums and housing estates were found to be infected by *T. cruzi* by examining fresh blood samples and smears stained with Giemsa, hemoculture, xenodiagnoses, and histopathology of opossums, as well as the examination of vector fecal samples.

The author suggested that the omnivorous/predatory diet of these marsupials, which feed on the reservoirs and vectors of *T. cruzi*, increases the probability of their becoming infected by this heteroxenous parasite. This, together with their synanthropic nature has enabled them to easily adapt to anthropically modified environments frequently invading areas inhabited by human beings. Furthermore, vectors showed positive, by immunodiffusion, for human and animal blood.

The tissue ubiquity and the pathology caused by *T. cruzi* obtained from *D. marsupialis* were determined in this host and experimental NMRI mice (23–26) (Table 1).

**Table 1:** **Histopathology in organs of white mice infected with isolates of *Trypanosoma cruzi* from urban *Panstrongylus geniculatus***.

Organ	Observations	Isolates			
		VP1	VP2	VP5	VP7
Heart	Diffuse myocarditis	XXX	XXX	XXX	
	Pancarditis		XXX		
	Myocyte destruction	XX	XXX	XXX	
	Abundance of amastigote and trypomastigote nests	XXX	XXX	XX	XX
	Fibroblast proliferation			X	
	Histiolymphomonocytic inflammatory infiltrate	XX	XXX	XX	XX
	Ganglionitis and periganglionitis	X			
	Neural edema and destruction	X			
Skeletal muscle	Histiolymphocytic myositis	XXX	X	XXX	XXX
	Abundance of amastigote and trypomastigote nests	XX	XX	X	XX
	Myocyte destruction	X		XXX	XX
	Fibroblast proliferation		X	X	
	Neuritis, perineuritis, perivascularitis	X			X
Duodenum	Inflammation of smooth muscle only, with:	X		X	X
	Abundance of small parasite nests	X	X	XX	
	Parasitization of myoenteric plexi	X		X	
Colon	Diffuse inflammation of smooth muscle only,	X	X	X	X
	With: abundance of small parasite nests	X	X	XX	X
	Parasitization of myoenteric plexi	X	X	XX	X
Liver	Scanty discrete inflammatory foci in parenchyma	X	X		X
	Foci of parenchymal necrosis			X	X
	Amastigote nests in gall bladder smooth muscle		X		X
Spleen	Hyperemia in red pulp		X	X	
	Scanty amastigotes in fixed macrophages of sinusoids	X			X
Pancreas	Amastigotes in acinous cells, Islets of Langerhans, and connective tissue	X	XX	XX	X
Lung	Small amastigote nests in peribronchial smooth muscle or arteriole walls	X	X	X	X
	Discrete inflammatory foci	X			X
Brain	Scanty parasite nests in microglial cells	X			
	Scanty parasite nests in white matter	X	X	X	X
	Parasite nests in cerebellum				XX
	Discrete inflammatory foci		X		
Bone marrow	Scanty amastigotes in fixed macrophages		X		

*X, moderate; XX, marked; XXX, intense*.

Deane et al. (27) described a unique property of *D. marsupialis*, whereby the *T. cruzi* morphotypes typically found in the intestines of its vectors, were also observed in the anal scent glands of this species. This property has been reported from wild *D. marsupialis* and *Didelphis albiventris* in both Brazil and Venezuela (28–30). In addition, the experimental colonization of *D. marsupialis* and *Lutreolina crassicaudata* glands was produced by s.c. injection of bug urine and fecal material (31–33).

Urdaneta-Morales et al. (34, 35) described this *T. cruzi* luminal cycle from naturally infected *D. marsupialis* captured in highly urbanized areas within the Caracas Valley. Numerous epimastigotes of different sizes, most undergoing binary division giving rise to rosettes, intermediate forms, and metacyclic trypomastigotes infective to mammals, were observed. These glands, thus, provide a favorable environment for the development of the *T. cruzi* vector cycle.

**Figure 2:**
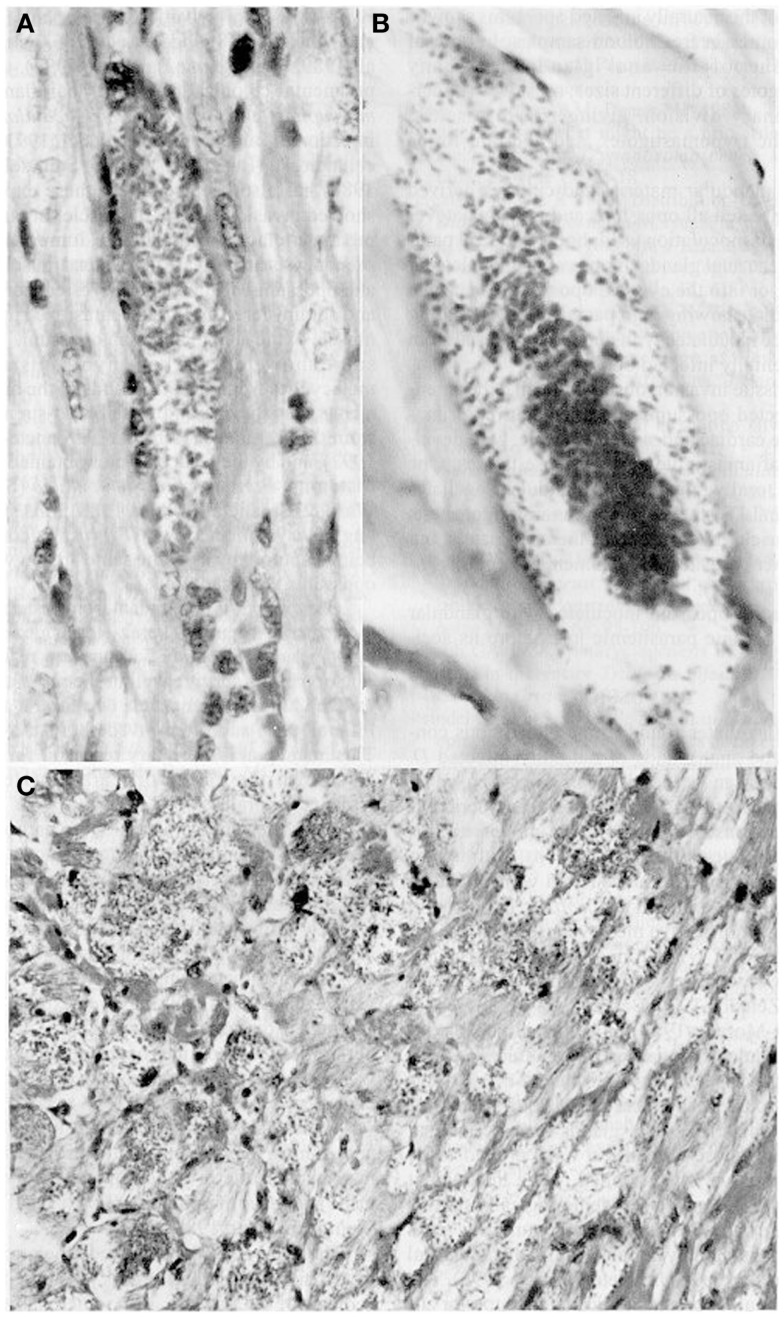
**Tissue sections showing pseudocysts containing amastigotes (H-E)**. **(A)** Heart of *Didelphis marsupialis* infected orally by glandular material cultured in LIT medium (400×; **(B)** muscle layer of anal gland of opossum, infected as above (1000×); **(C)** heart of mouse infected intraperitoneally by glandular material from a naturally infected opossum (400×).

Furthermore, glandular material inoculated s.c., i.p., or instilled orally and ocularly in healthy opossums and white mice caused parasitemias with the pleomorphic forms typical of the parasites as well as intracellular parasitism with the multiplication of amastigotes in cardiac and skeletal muscle (Figure 2). Glandular metacyclics from LIT (liver infusion tryptose) cultures inoculated into these mammals gave the same results. Infection caused 100% mortality in the mice, whereas all the opossums survived. Nymphs of *R. prolixus* fed on both hosts showed infective metacyclics in the feces (34, 35).

**Figure 3:**
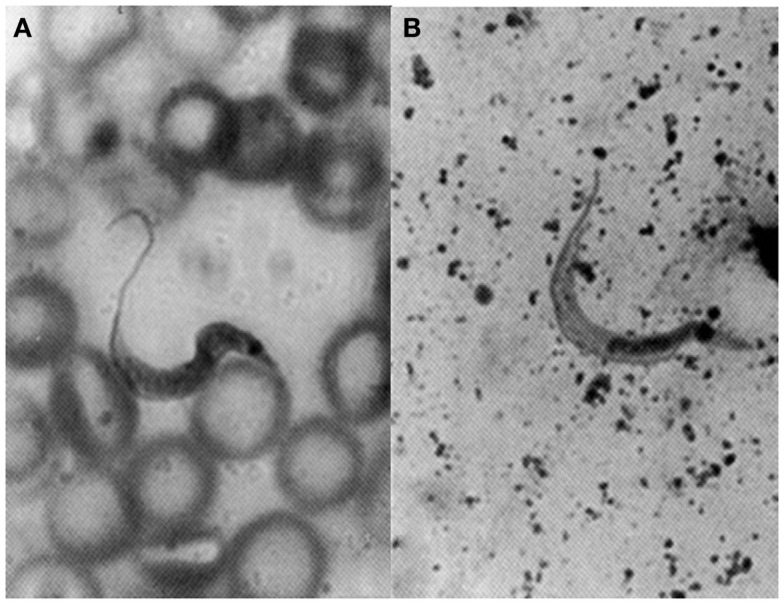
**Flagellate stages of *Trypanosoma cruzi***: **(A)** stout bloodstream trypomastigote from naturally infected *Rattus rattus* (Giemsa, 1400×); **(B)** metacyclic trypomastigote from feces of *Rhodnius prolixus* used for xenodiagnosis of naturally infected *R. rattus* (Giemsa, 1400×).

**Figure 4:**
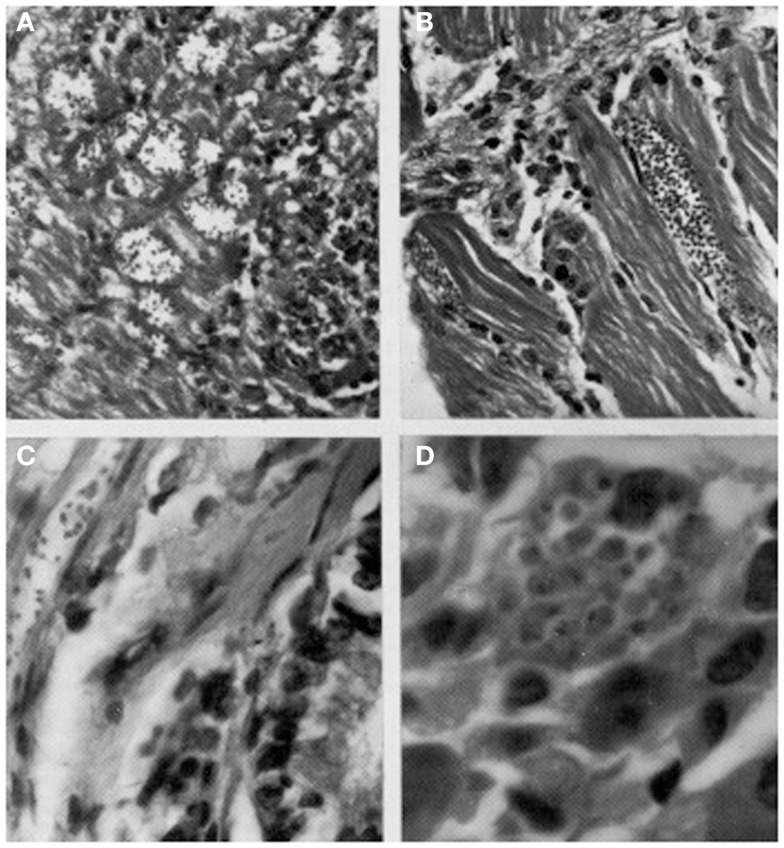
**Histological sections showing pseudocysts of *Trypanosoma cruzi* with amastigotes in (A) cardiac tissue of naturally infected *Rattus rattus* (H-E; 560×); (B)** skeletal muscle of naturally infected *R. rattus* (H-E, 560×); **(C)** smooth muscle fiber from the colon of an experimentally infected mouse (H-E, 960×); **(D)** acinar cell of pancreas of experimentally infected mouse (H-E, 1400×).

Herrera and Urdaneta-Morales (36) captured 37 *Rattus rattus*, one *R. norvegicus*, and nine *Mus musculus* in urbanized areas (Colinas de Bello Monte, Los Chorros, El Cafetal, Las Acacias, San Román, Parque del Este and Caricuao) in Caracas. Of these, conventional examinations of fresh blood and smears stained with Giemsa, and xenodiagnosis revealed the presence of *T. cruzi* stages in two *R. rattus* individuals (Figure 3). Tissue sections from *R. rattus* showed numerous pseudocysts with amastigotes in the heart as well as moderate parasitism of the skeletal muscle and smooth muscle of the duodenum. All mice inoculated with xenodiagnosis’ bug fecal material showed a moderate pattern of tissue tropism in the same organs as in well as in the colon and pancreas (Figure 4). *Trypanosoma* (*Herpetosma) lewisi* was also detected in infected rats, but not in the other rodents examined.

Reyes-Lugo and Rodríguez-Acosta (37) found *P. geniculatus* colonizing the interior of a well-built house in an area of transition between cloud forest and humid montane woodland in the mountainous region of Hoyo de la Puerta (Miranda state) on the outskirts of Caracas. A total of 20 *P. geniculatus* specimens in all stages of development gorged with blood were shown to be infected by *T. cruzi*. The authors also found *R. rattus* in several tunnels connecting the inside floor of the house with the outdoors, in which several *P. geniculatus* individuals and their eggs were found. The authors suggested that human activities have led to the disappearance of the natural habitats of this triatomine and with them its food sources, thus favoring its domiciliation. This situation has been described by Reyes-Lugo (38) in a further eight localities in the center-north of Venezuela, including the Caracas Valley.

**Figure 5:**
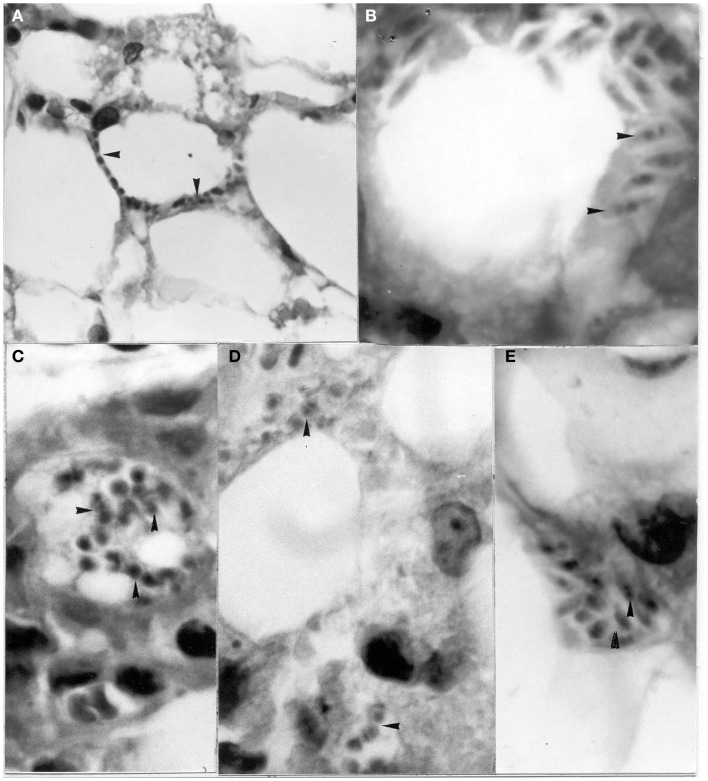
**Amastigotes and intermediate stages (arrows) of *Trypanosoma cruzi* in (A,B) perifery cytoplasm of an adipocye**; **(C)** cytoplasm of immature adipocite (preadipocite); **(D)** intercellular substance in connective adipose tissue; **(E)** parasitized macrophage located between uninfected adipocytes (H-E; 1400×).

**Figure 6:**
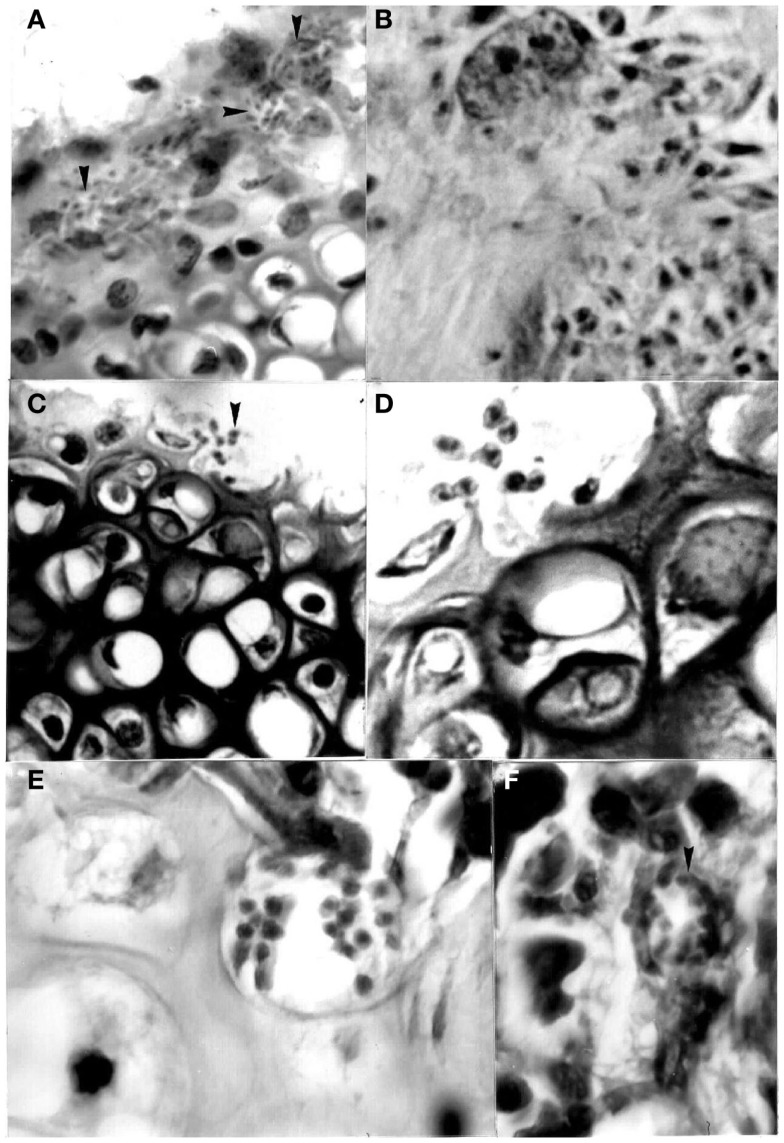
**Pseudocysts with stages (arrows) of *Trypanosoma cruzi* in sternum of mice experimentally infected with different isolates**. **(A)** Perichondrium with nests showing amastigotes and intermediate stages in chondroblasts (400×; HE); **(B)** same stages in stroma of perichondrium and in the cytoplasm of a macrophage (1000×; HE); **(C)** and **(D)** two broken chondrocytes with several amastigotes and one flagellated stage (400× and 1000×, respectively; HE); **(E)** several amastigotes in a osteocyte (1000×; HE); **(F)** pseudocyst with amastigotes in the bone marrow (1000×; HE).

**Figure 7:**
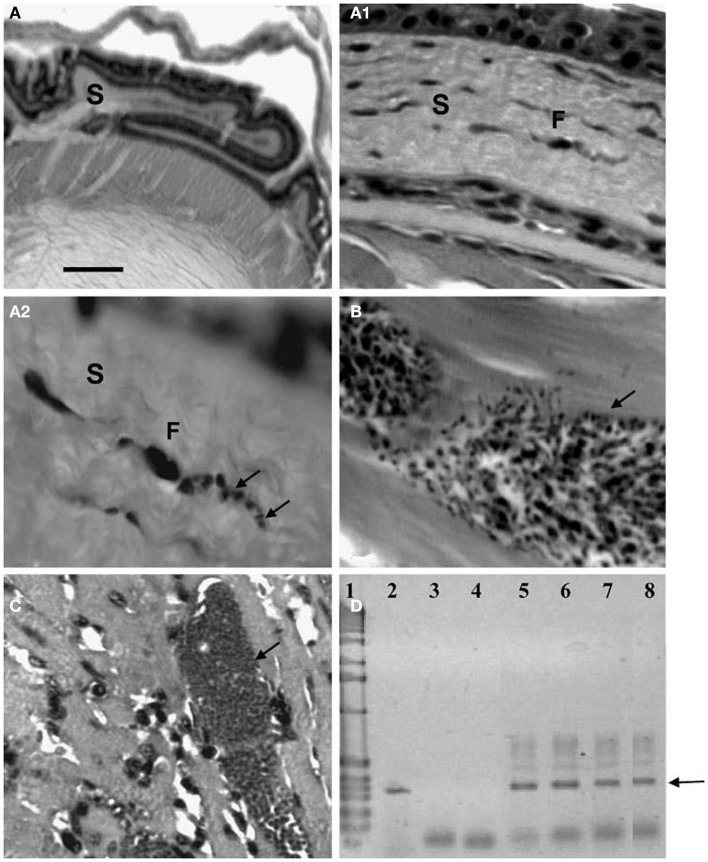
**Histological and molecular parasitism in NMRI mice experimentally infected with different isolates and strains of *T. cruzi***. **(A – A2)**. Sequence of microphotographs with amplification of a nest of amastigotes in a fibroblast (F; arrows; 40×, 400×, and 1000×, respectively) of corneal stroma (S); **(B)** trypomastigote nest in thigh skeletal muscle (arrow); **(C)** amastigote nest in heart muscle (arrow); **(D)** amplification of the 330-bp fragment from the conserved regions of kDNA (arrow) extracted from ocular tissues of experimentally infected NMRI mice in 2.5% agarose gel electrophoresis (ethidium bromide stain): Lane 1 1-kb ladder molecular marker (Gibco BRL Life Technologies), lane 2 nude *T. cruzi* DNA, lane 3 negative PCR control, lane 4 MRAT/VE/1996/CO22 isolate, lane 5 MHOM/BR/1950/Y strain, lane 6 MHOM/VE/1970/EP isolate, lane 7 MDID/BR/1999/M1 isolate, and lane 8 MDID/VE/1995/CO79 isolate.

Xenotransplantations have demonstrated zoonotic infections produced by viruses, bacteria, protozoa, fungi, and helminths, thus showing their importance as risk factors for these diseases (20). Using a mouse model, the possibility of the transfer of *T. cruzi* was determined in organs often used during these procedures. Isolates from *D. marsupialis* and *R. rattus* captured in Caracas were inoculated in adipose, bone-cartilage, and eye tissue, observing the intracellular presence of the parasite in all cases (39–41) (Figures 5–7). This constitutes an alternative transmission pathway, whereby natural *T. cruzi* intracellular multiplication could be enhanced in immunosuppressed hosts. Isolates of *T. cruzi* stages found in the eye tissue of mice produced an electrophoretic band pattern that identified the parasite as TcI (ZI).

**Figure 8:**
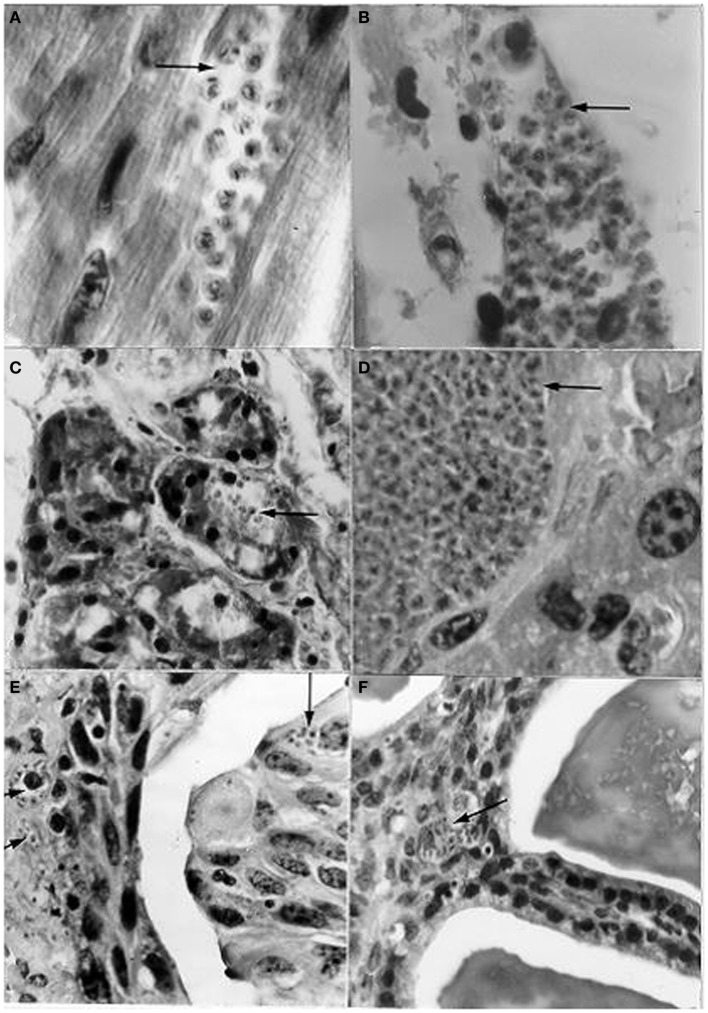
**Histological sections from albino mice intravaginally instilled with a strain of *Trypanosoma cruzi* from *Rattus rattus*, and scrotally inoculated with a strain from a human patient, showing nests (arrows) with amastigotes in (A) cardiac tissue; (B)** skeletal muscle; **(C)** pancreas (acinus); **(D)** liver; **(E)** urinary bladder (epithelium very close to the lumen and *lamina propria*); **(F)** seminal vesicle (mucosa close to the lumen). [**(A,B,D)**: l400×; **(C,E,F)**: 950×; H-E].

Based on several references cited by Zeledón (42) regarding the presence of *T. cruzi* trypomastigotes in opossum (*D. marsupialis)* and mouse urine, together with the suggestion of Dias (43) that the presence of these stages in the chagasic female’s menstrual blood could enable transmission through coitus, Herrera and Urdaneta-Morales (44) carried out the following investigation: the blood of mice infected with *T. cruzi* isolates from *R. rattus* was instilled into the urinary meatus (females) and penis (males) of healthy mice, and a *T. cruzi* isolate from a human being was inoculated into the scrotum. The genital instillations resulted in the invasion of the heart, skeletal muscle, duodenum, pancreas, sternum, ovary, testis, and vas deferens. In addition, scrotal inoculation caused the invasion of the liver, spleen, lung, kidney, urinary bladder, and seminal vesicle (mucosa close to the lumen) (Figure 8).

Carrasco et al. (21) established that there is a high risk of the transmission of Chagas’ disease in the Caracas Valley due to the contact between *P. geniculatus* and human beings. Natural infection by *T. cruzi* was determined by examinations of fresh and stained stools. In addition, the random amplification of polymorphic DNA for parasite identification and group typing, and a dot-ELISA test to identify the gut content of the triatomine bugs showed that 66% of the 88 triatomines studied were infected by TcI, of which 60% reacted positively to human antiserum. The relationship between the percentages of bugs with fecal contents reactive to human antiserum and those reactive to all the antisera used was 98%, while approximately 41% of the bugs fed on human beings were infected by *T. cruzi*.

The first proven cases of the transmission of *T. cruzi* to human beings occurred in urbanized areas within the Caracas Valley (Chacao municipality and Antímano parish). These outbreaks represent two of the most numerically important cases of orally acquired Chagas disease in Latin America to date. A total of 124 patients in the acute phase of infection were characterized by their clinical symptoms, electrocardiograms, immunoenzymatic tests, indirect hemagglutination, and PCR. Examinations of blood, hemoculture, and inoculation in a mouse model were also performed. Polluted guava juice was statistically shown to be the only source of infection and the person who prepared the juice was infected with *T. cruzi*. The presence of infected *P. geniculatus* and *R. rattus* was confirmed in the slum where this individual lived (45, 46).

The capture of two specimens of *Panstrongylus rufotuberculatus* infected with *T. cruzi* inside houses in the town of El Hatillo, close to Caracas (originally a forested area but now completely urbanized), was reported by Zavala-Jaspe et al. (47). One of the isolates was characterized by the following: parasitological examinations of the intestinal contents, infection of a mouse model, hemoculture, xenodiagnoses, and *in vivo* and *in vitro* metacyclogenesis producing morphotypes characteristic of the parasite; infection by *T. cruzi* was confirmed by PCR.

All of these chronologically described results constitute important epidemiological risks for public health in this capital city.

## Dynamic of Emergent Diseases: Transmission Pathways-Disease Transmissions

Until the end of the 80s, it was thought that emergent diseases had been eradicated or that they were limited to underdeveloped areas. This was due to the fact that the biomedical control programs were abandoned, which resulted in a huge spate of emerging diseases. The situation was aggravated by the failure to isolate or identify the pathogens responsible, coupled with the difficulty of characterizing the symptoms due to their (in the majority of cases) extensive pre-patency and subclinical nature. Research into the global occurrence of important outbreaks led to the conceptualization of the causes of the emergence of these diseases (15, 19). In both excellent reviews of the many risk factors that influence the emergence of infectious diseases according to their pathogens, an estimated 75% of these diseases, principally viral, bacterial and parasitic, and possibly vector-borne, have emerged over the past two decades from a wildlife source.

Given that each reservoir and vector species occupies a specific ecological niche and shows a particular behavior pattern, the physical and biological environments should be maintained in a dynamic equilibrium with human populations and society in order to prevent the emergence and spread of new diseases. It is important to realize that the emergence of these diseases is most likely to occur after changes to the components of this dynamic equilibrium. Consequently, zoonoses (including the parasitic ones) and particularly emergent zoonoses have sparked interest in the international scientific community. This is due to their increasing importance to human and animal health as they cause high indices of morbidity and mortality in both rural and urban areas and in endemic and non-endemic areas, with huge repercussions for the economies and health of the countries affected (5, 48–52).

Human beings, in their struggle for survival, have profoundly modified the natural environment, adapting it to their needs as they colonize different landscapes and habitats, all of which provide natural niches for pathogens responsible for zoonotic infections. These can derive from, and become emergent in, many different environments: wild, rural, regional-urban, and even global-urban. These niches house an enormous number of vertebrates and invertebrates that, in turn, host a huge variety of macro- and micro-pathogens; the risks of transmission to human beings are obvious (5).

Anthropogenic changes (environmental changes, deforestation, reforestation, road construction, urban growth, trade, translocation, and keeping of exotic pet, ecotourism, consumption of contaminated water or raw or undercooked food in order to retain its flavor and nutrients) are those that have proven to cause the most stress on the balance between the physical and biological environments intrinsic to the pathogen-host-vector association.

The key factor for the unprecedented levels of these emergent diseases is the synergism between two circumstances, the globalization of human activities with the exponential increase in the population growth rate causing progressive deterioration of the relationship between human and environmental health, and therefore modification and destruction of the natural niches of reservoirs, vectors, and pathogens. Unprecedented increases in the incidence of infectious emergent diseases and the extinction of species with the resulting loss of biodiversity are examples of this. Immune pressure and the abuse in antibiotic use also cause highly frequent changes in pathogens that are expressed as mutations in their virulence, pathogenicity, and genetic structure. Another important characteristic worthy of mention is the generalist nature of the pathogens that affect human beings: those that are capable of infecting multiple hosts, especially when they belong to more than one order, are highly likely to infect human beings. An excellent example of this type of pathogen is *T. cruzi* that, as already mentioned, parasitizes species of mammals from 7 orders and triatomines from 15 genera (10, 15–20, 48–61).

## Neglected Zoonoses

The critical situation regarding infectious diseases that persists in many indigenous populations in several continents must be highlighted. These zoonoses have a higher prevalence in regions between latitudes 35°N and 35°S, with altitudes below 2200 m and temperatures ranging from 15°C to 40°C. These populations are some of the world’s poorest, most anonymous and ignored. They have been subjugated by foreign powers that “discover” them, conquer and enslave them; they have suffered unjust discrimination and political and economic exclusion. Multinational corporations have, among many other things, denied them their rights to education, their sources of cultural and physical survival, their traditional knowledges, the ownership of some of the most biologically diverse territories in the world thus producing local or global extinctions of species, and their languages, in short, their ethnic cultural identity.

Emerging diseases are considered as “neglected” because the huge investments resulting from The Millennium Declaration, adopted by the United Nations in September of the year 2000 and applied at a large scale to projects for the prevention and/or control of HIV/AIDS, malaria and tuberculosis (the “big three”), have not up until now been used for combatting them. Many scientists, institutes for health and pharmaceutical companies continue to ignore and exclude them. The argument put forward for this is that there is no reliable information about the health burdens of these “low hanging fruit” populations. This lack of knowledge has led to a reduction or elimination of funding and research within these marginalized communities. All this, in spite of the fact that millions of people from Sub-Saharan Africa, Asia, Latin America, and the Caribbean continue to suffer diseases at least as (if not more) serious as the three mentioned (62–68).

## Programs for the Prevention and Control of Zoonose

The great diversity of risk factors that produce the emergence, re-emergence and, in many cases, persistence of zoonoses caused by pathogens that are already known, as well as those that have only recently been characterized, or have undergone changes in their bio-ecological characteristics in poor communities are particularly notable in countries governed by strong socio-politico-religious groups, as is the case for Africa, Asia, and also Latin American and Caribbean countries whose populations are indigenous or of African descent.

By other hand, most of the alterations in the behavior, cognitive or psychosocial patterns of the indigenous populations infected with zoonoses are, in general, similar even between communities from different continents, it is appropriate to mention them here. The *per se* knowledge that they are ill, or if they are told that they have a disease of this nature, produces symptoms that begin with slight depression, before developing into anxiety, stigma, and alexithymia. Their concern that the disease will worsen, the impossibility of finding a cure and their fear that they will infect others, distances them from their family circle and friends, while the weakening of their immunological systems only serves to worsen their condition (68–70).

Currently (63–65), a comprehensive framework model is being applied in some continents with promising results. This model attempts to control some diseases and reduce poverty with a view to returning the basic rights of these populations (equality, ethical treatment, and health) stolen from them. According to specialists, a key strategy for minimizing the deficiencies in funding is to try to increase the commitment of the family and local community so that they themselves help to prevent, control, and even eliminate zoonoses through feasible and economic methods. Some examples of such community-based approaches are Community-Led Total Sanitation (CLTS), which has completely eliminated open defecation resulting in a massive reduction in the incidence of enteric diseases; controls in China for reducing the rate of transmission of *Schistosoma japonicum* from infected bovines and human beings to snails; management of sleeping sickness in Uganda caused by *T. rhodesiense* by chemotherapy of bovine reservoirs en masse and the use of insecticides for the control of the insect vectors.

Molyneux and Malecela (66) drew up a set of macro-priorities and recommendations with the idea of clarifying these objectives as listed below (Table 2). These authors emphasize the scarce knowledge that clinicians and policy makers, general speaking, have when they diagnose for example a zoonosis with no specific symptoms such as Brucellosis, Leptospirosis, Rickettsiosis, or Q fever, as Malaria, as well as their ignorance of the possibility that zoonotic infections can, in the long term, cause cancer (Trematodiases) or Neurocysticercosis. All this as a consequence of the absence of microbiological and molecular tools in laboratories and hospitals, due to the financial limitations they suffer; further evidence of the sad neglect these populations face.

**Table 2:** **Macro research priorities identified by DRG6 (Disease Reference Group – WHO UNDP World Bank Special Programme)**.

There is a need to develop a comprehensive methodology for calculating the societal burden of disease attributable to zoonoses recognizing that a high proportion of the population of rural (and often urban) populations in least-developed countries depends on livestock. More studies are required to generate data on the costs, cost-benefits, and cost effectiveness of interventions for endemic zoonoses. Such studies should also incorporate the economic effect of animal disease as an indirect contributor to poverty through its impact on nutrition, loss of meat and milk products, and livestock as a capital asset. There is a need for operational and systems research to identify reasons for the limited communication and interaction between the key sectors – health, agriculture, and livestock – particularly in countries where a large proportion of the population is dependent on livestock. There is a need to evaluate effective community-based approaches and interventions for zoonotic diseases, drawing on the experience and success of initiatives for water and sanitation improvements, mass drug delivery, and community-based health care. Experiences from separate initiatives in different geographic and epidemiological settings need to be evaluated to ensure that such experiences are amplified and synergized, with potential for integration between programs. Investing in systems for the collection of reliable data on disease/infection incidence and prevalence from both veterinary and medical sectors is recognized as a priority, both for the measurement of disease burden and the evaluation of control measures. Investment in endemic zoonoses in least-developed countries would provide multiple benefits not only by improving the health and livelihoods of marginalized communities but also by reducing threats and enhancing the response capacity for emerging zoonoses that pose a threat to the global community. Effective lessons are often best learned by the implementation of strategies (such as the onchocerciasis control program), with research to evaluate factors leading to success measured by effectiveness and cost-effectiveness embedded within program implementation. As endemic zoonoses disproportionately affect impoverished and marginalized populations, investments need to be specifically targeted to overcome barriers to health care in these communities, including isolation, population movement or migration, social or political unrest, and conflict.

With this in mind, strategies that should be developed include primary health education in conjunction with the health sectors of competent indigenous organizations; the translation of books into indigenous languages, training for indigenous health workers, teachers and community leaders with an emphasis on teaching about the main risk factors, and the time of year these are most prevalent; constant entomological and serological monitoring, after training, by community leaders; the implementation of vector control methods, such as indoor residual spraying pyrethroid and improving housing; safe preparation of food for its immediate consumption or transport; safe water for drinking and basic sanitary conditions and the monitoring and treatment of infected individuals. In addition, surveillance and the use of drugs and vaccines, research in natural products; clinical and epidemiologic research; use of electron microscopy, genomics/bioinformatics, and applied biotechnology should be available to ensure healthier lives. All these aspects should occur within the context of environmental sustainability, and all are necessary in the regions where most of the zoonoses are found. There is an urgent need for a concerted effort by disciplines such Ecology, Epidemiology, Medical Entomology, Human and Veterinary Medicine, Public Health, Environmental Studies, Immunology, Microbiology, Pharmacology, Social and Political Studies, and Anthropology (62, 69–77).

We list the following pathogens, many of them emergent–reemergents with the potential to cause neglected pandemic zoonoses, based on the definitions of emergent diseases already given in this review: Human immunodeficiency virus (VIH), Ebola virus, Dengue virus, Simian immunodeficiency virus, Hanta virus, Hendra virus, Nipah virus, Menangle virus, West Nile virus, Avian influenza A H5N1 virus, Monkeypox virus, SARS virus, Rift Valley fever virus, Junin virus (Argentine hemorrhagic fever), Cercopithecine herpesvirus (Meningoencephalitis virus), Australian bat lyssavirus, *Rickettsia africae (*African tick bite fever), *Ehrlichia canis* (Leukocytic Rickettsia, human being, dogs), *Vibrio cholerae* (cholera), *Borrelia burgdorferi* (Lyme borreliosis), *Leptospira interrogans* (Icteric leptospirosis), *Treponema pallidum* (Syphilis), *Mycobacterium tuberculosis* (tuberculosis), *M. ulcerans* (Buruli ulcer), *M. leprae* (leprosy), *Chlamydia trachomatis* (human blinding trachoma; sexual disease), *C. psittaci* and *C. pecorum* (birds, mammals), *Clostridium difficile* and *Staphylococcus aureus* (antibiotics resistant), *Brucella suis* (wild boar brucellosis), *Salmonella* sp. (serotype enteritidis), *Coxiella burnetii* (Q fever), *Campylobacter jejuni*, and *Listeria monocytogenes* (gastroenteritis by contaminated food), *Cryptosporidium* spp. (waterborne disease human being, animals), *Escherichia coli* O157:H7 (enterohemorragic strain, Toxic shock syndrome by contaminated food; verotoxine production), *Leishmania* (*Leishmania)* spp. (muco-cutaneous and visceral leishmaniasis), *T. cruzi* (Chagas’ disease), African *Trypanosoma, Plasmodium* spp. (Malaria); *Ascaris* spp., *Necator americanus*, *Ancylostoma* spp., *Trichuris* spp. (whipworms), *Strongyloides* spp. (Geohelminths, digestive, pulmonary system infections), *Dracunculus* spp. (Guinea worm disease; human being, animals), *Onchocerca* spp. (river blindness), *Wuchereria bancrofti* (lymphatic filariasis, “elephantiasis”) (Nematelminthes infections); *Schistosoma* spp. (urinary and intestinal schistosomiasis or bilharzia), *Clonorchis sinensis* (liver and bile duct cancer), *Opisthorchis viverrini* (cholangiocarcinoma: gall bladder cancer), *Fasciola hepatica* (liver fluke disease, human beings, ungulates), *Paragonimus westermani* (acute lungs infection) (Platyhelminthes, Trematoda infections); *Tenia solium* (Neurocysticercosis) (Platyhelminthes, Cestoda infection); and *Sarcoptes scabiei* (mite, scabies) among others (13, 15, 16, 52–54, 57, 60). This list of emergent–reemergent pathogens should, like any other, be periodically revised and updated given the dynamics of the factors that determine changes in its transmission pathways.

Following on from this, it is appropriate to discuss the following considerations as a consequence of the permanent rise in industrialization and urbanization in Latin America and the Caribbean Region, the transmission of several infectious diseases, has increased dramatically (4, 5, 20, 55, 64, 69, 78–83). Its diagnosis, however, can be delayed for several months depending on the technique used. This obviously delays treatment causing the patient’s condition to worsen. The fact that *T. cruzi* and its vectors can remain viable in food over long periods of time only exaggerates this problem. Nevertheless, these situations can be averted with the use of newly developed molecular tools, which, together with reference datasets, have shortened the diagnosis time to only a few days (45, 46, 84). It is to be hoped that in the future, some of the risk factors of these diseases will be at least qualitatively and quantitatively reduced, if not eliminated. Several of these measures could seem utopic because of the small attention they have traditionally received, but it would be a mistake not to mention them. The current healthcare system, although insufficient, should at least be used to improve the situation of the poorest communities while other tools are being developed. The need for investigation into the use of drugs to combat the infections, while minimizing the risks of the evolution of pathogen resistance to them, is obvious (85).

The investigations undertaken in the Caracas Valley, presented and discussed using a bio-eco-epidemiological approach, show that Chagas’ disease is most certainly an emerging urban zoonosis and underline the high risks of infection for human beings and their domestic animals.

The following is a summary of the most important epidemiological aspects of the public health situation regarding the transmission of Chagas’ disease by *D. marsupialis* in the Caracas Valley, which taken together create conditions of permanent risk from zoonotic infection by *T. cruzi* in areas with or without the presence of vectors: the adaption of *D. marsupialis* to city environments, from slums to economically wealthy urbanized zones; the capacity this marsupial has to infect mammals with metacyclic trypomastigotes that develop in the lumen of the anal scent glands; the violent ejection of fluid from the scent glands in response to threat and the proximity of these glands to the rectum and the urogenital organs, thus providing a means by which the feces and urine can become contaminated with the glandular fluid containing the metacyclics, leading to the possibility of infection through the consumption of spoiled food and drink.

The finding that contact between *T. cruzi* bloodstream trypomastigotes and genital mucosa can produce blood and tissue infections through the formation of pseudocysts with amastigote stages close to the lumen of the urinary bladder and seminal vesicle suggests an alternative possibility for the transmission of this zoonosis; when the cysts rupture, they could liberate the parasites either into the genital secretions or urine and thus to the exterior.

The fact that *R. rattus* may also act as a reservoir for *T. cruzi* and the evolution of the adaption of *P. geniculatus* and *P. rufotuberculatus* to human dwellings increase and geographically widen the risks for the transmission of Chagas’ disease in the Caracas Valley.

The infection of adipose, bone-cartilage, and eye tissues by *T. cruzi* demonstrates their role as important micro-reservoirs of this parasitic flagellate and reveals yet another transmission pathway during organ transplants in immunosuppressed patients.

This review was initially undertaken in response to the risks of transmission brought about by the close contact between *D. marsupialis*, *P. geniculatus*, and human beings along the whole of the Caracas Valley, and the report (for the first time) of 124 acute cases of proven *T. cruzi* transmission to inhabitants in a totally urbanized sector of this capital city, as well as important results from other investigations carried out in areas encompassing the poorest slums to zones of high economic value. The permanent and high levels of industrialization and the migration of people from endemic regions of Venezuela and other countries can only further increase the probability of the emergence of this zoonosis.

## Conflict of Interest Statement

The author declares that the research was conducted in the absence of any commercial or financial relationships that could be construed as a potential conflict of interest.
